# Evaluation of Herb–Drug Interaction Between Danshen and Rivaroxaban in Rat and Human Liver Microsomes

**DOI:** 10.3389/fphar.2022.950525

**Published:** 2022-07-19

**Authors:** Xu Wang, Jingjing Fa, Yuanjin Zhang, Shengbo Huang, Jie Liu, Junqing Gao, Lina Xing, Zongjun Liu, Xin Wang

**Affiliations:** ^1^ Department of Cardiology, Putuo Hospital, Shanghai University of Traditional Chinese Medicine, Shanghai, China; ^2^ Shanghai Key Laboratory of Regulatory Biology, Institute of Biomedical Sciences, School of Life Sciences, East China Normal University, Shanghai, China; ^3^ Putuo Clinical Medical School, Anhui Medical University, Shanghai, China

**Keywords:** Danshen, rivaroxaban, herb-drug interaction, metabolism, cytochrome P450 (CYP)

## Abstract

The combination of *Salvia miltiorrhiza* (Danshen) and rivaroxaban is a promising treatment option in clinical practice in China, but the herb–drug interaction between Danshen and rivaroxaban remains unclear. Therefore, this study aims to reveal the interaction between Danshen and rivaroxaban. We not only investigated the inhibitory properties of Danshen tablet on rivaroxaban metabolism in rat and human liver microsomes but also evaluated the inhibitory effects of Danshen tablet and its eight active components (dihydrotanshinone I, tanshinone I, tanshinone IIA, cryptotanshinone, danshensu, salvianolic acid A, salvianolic acid B, and salvianolic acid C) on cytochrome P450 (CYP) enzymes. The results showed that Danshen tablet potently inhibited the metabolism of rivaroxaban in rat and human liver microsomes. In the CYP inhibition study, we found that dihydrotanshinone I, the active component of Danshen tablet, potently inhibited the activities of rat CYP3A and CYP2J, with IC_50_ values at 13.85 and 6.39 μM, respectively. In further inhibition kinetic study, we found that Danshen tablet is a mixed inhibitor in rivaroxaban metabolism in rat and human liver microsomes, with the *K*_i_ value at 0.72 and 0.25 mg/ml, respectively. In conclusion, there is a potential interaction between Danshen tablet and rivaroxaban. Danshen tablet inhibits the metabolism of rivaroxaban, which may be because its lipid-soluble components such as dihydrotanshinone I strongly inhibit the activities of CYP enzymes, especially CYP3A and CYP2J. Therefore, when Danshen tablet and rivaroxaban are used simultaneously in the clinic, it is necessary to strengthen the drug monitoring of rivaroxaban and adjust the dosage.

## Introduction

The combination of traditional Chinese and Western medicine can improve drug efficacy and increase patient tolerance. However, the risk of herb**–**drug interactions (HDI) increases. Inhibition/induction of the drug-metabolizing enzymes is an important reason for the HDI (Li et al., 2019). Cytochrome P450 (CYP) enzymes as the important phase I drug-metabolizing enzymes play an important role in HDI (Li et al., 2019). CYP enzymes are divided into 17 families and many subfamilies, among which CYP1, CYP2, and CYP3 account for about 70% of total CYP (Toselli et al., 2016).

Rivaroxaban, a direct oral anticoagulant (DOAC), inhibits factors Ⅹa, thus preventing the blood clotting cascade and inhibiting thrombosis (Ajmal et al., 2021). Rivaroxaban is used in clinical practice to treat venous thromboembolism after elective hip and knee arthroplasty (Huang et al., 2018). It can also prevent stroke and systemic embolism in patients with non-valvular atrial fibrillation (NVAF) (Desai et al., 2020). In addition, the European Medicines Agency (EMA) has approved rivaroxaban for the prevention of thromboembolic events in patients with acute coronary syndrome (ACS) (Cowie et al., 2020). Rivaroxaban is metabolized by CYP3A4, CYP2J2, and non-enzymatic hydrolysis, and the proportions are about 18, 14, and 14%, respectively (Mueck et al., 2013; Cheong et al., 2017; Wang et al., 2021). It has been reported that ketoconazole significantly inhibits the metabolism of rivaroxaban and increases its exposure *in vivo*, which may be caused by liver CYP inhibition (Foerster et al., 2020).

Danshen tablet is effective for cardiovascular and cerebrovascular diseases and is widely used around the world, especially in China (Zhang et al., 2020). Danshen tablet contains a variety of aqueous soluble and lipid-soluble components. Water-soluble components, such as danshensu and salvianolic acid A and B, have many pharmacological activities, including antioxidant and cytoprotective effects (Wang and Yeung, 2011). Lipid-soluble components, such as tanshinone I, tanshinone IIA, dihydrotanshinone, and cryptotanshinone, possess pharmacological properties such as promoting blood circulation and antibacterial and anti-inflammatory (Wang and Yeung, 2012; Guo et al., 2020). Danshen tablet is characterized by complex composition and complicated mechanism. Its active components can affect the expression level and activity of CYP (Wang et al., 2016; Meng et al., 2021). For example, our previous studies reported that dihydrotanshinone is a non-competitive inhibitor of CYP3A4 (Wang et al., 2010).

As we all know, Danshen and rivaroxaban are always simultaneously used to treat heart disease. However, the interaction between Danshen and rivaroxaban remains unknown. From the perspective of patient safety, we should pay close attention to HDI to formulate a reasonable treatment plan and avoid adverse reactions. In this study, we explored the interaction and mechanism between Danshen tablet and rivaroxaban in rat and human liver microsomes. Clinical practitioners are expected to benefit from these findings.

## Materials and Methods

### Chemicals and Reagents

Dihydrotanshinone I (purity > 97%), tanshinone I (purity > 98%), tanshinone IIA (purity > 98%), cryptotanshinone (purity > 98%), danshensu (purity > 99%), salvianolic acid A (purity > 99%), salvianolic acid B (purity > 99%), salvianolic acid C (purity > 98%), and rivaroxaban (purity > 99%) were purchased from TaoSu Biochemical Technology Co. Ltd. (Shanghai, China). Tris (hydroxymethyl) aminomethane hydrochloride (Tris-HCl), β-nicotinamide adenine dinucleotide phosphate (NADP), glucose 6-phosphate dehydrogenase (G6PDH), glucose 6-phosphate (G6P), phenacetin, bupropion, tolbutamide, dextromethorphan, chlorzoxazone, astemizole, and midazolam were purchased from Sigma Chemical Co. (St. Louis, MO, United States). Mebendazole (internal standard) was obtained from Aladdin Industrial Co. (California, United States). Danshen tablet was purchased from Shanghai Lei Yun Shang Pharmacy Co., Ltd. (Shanghai, China). Pooled rat liver microsomes (RLM) and human liver microsomes (HLM, *n* = 25) were obtained from the Research Institute for Liver Diseases Co., Ltd. (Shanghai, China) and stored at −80°C until use. Methanol and acetonitrile (HPLC grade) were purchased from Fisher Chemicals (Leicester, United Kingdom). Formic acid and ammonium formate (HPLC grade) were purchased from TEDIA Company, Inc. (Ohio, United States).

### Equipment and Operating Conditions

In this study, an Agilent 1290 LC system consisting of a binary pump, a degasser, an autosampler, and a thermostatic column compartment was coupled with a 6470 triple-quadrupole mass spectrometer (Agilent Technologies, United States), which was equipped with an Agilent Jet Stream electrospray ionization (ESI) source and operated with Agilent Mass Hunter version 9.0.9037.0 software (Agilent Technologies, United States). Chromatography separation was performed on a Phenomenex Kinetex XB-C18 column (100 × 3.00  mm, 2.6 μM) protected by a Phenomenex C18 guard column (Torrance, CA, United States).

For rivaroxaban analysis, the LC-MS/MS method was implemented as described in the previous reports (Wang et al., 2020; de Oliveira et al., 2021). In brief, the mobile phase consisted of solvent A (1 mM ammonium formate in water) and solvent B (0.1% formic acid in methanol) using gradient elution at a flow rate of 0.3 ml/min. The following stepwise gradient elution program was used: 50% B (0–2 min), 50–70% B (2–6.5 min), and 70–50% B (6.5–8 min). The temperature of the column oven was maintained at 30°C, and the injection volume was 3 μL. The positive ion ESI mode was used to monitor ion transitions of *m/z* 436.1→144.9 for rivaroxaban under multiple reaction monitoring (MRM) analysis. The gas temperature was 250°C, and the gas flow rate was 10 L/min. Other parameters of ion source were as follows: nebulizer gas, 35 psi; sheath gas temperature, 350°C; sheath gas flow, 11 L/min.

For CYP substrate metabolite detection method, the optimal gradient mobile phase included solvent A (0.1% formic acid in water; v/v) and solvent B (0.1% formic acid in acetonitrile; v/v), with a flow rate of 0.3 mL/min. The best elution condition was as follows: 10% B (0–2.2 min); 10–90% B (2.2–8.5 min); 90–92% B (8.5–9 min); 92% B (9–9.3 min); 92–10% B (9.3–9.6 min); 10% B (9.6–11.5 min). The temperature of the column oven was kept at 30°C. In an ESI mode, simultaneous scanning of positive and negative ions was performed by polar switching. Ions were detected under the multiple reaction monitoring (MRM) mode. The gas temperature was 250°C, and the gas flow rate was 10 L/min. Other parameters of ion source were nebulizer gas, 35 psi; sheath gas temperature, 350°C; sheath gas flow, 11 L/min. LC-MS/MS analysis for all analytes has been introduced in our previous method and validation (Chen et al., 2016; Tang et al., 2017).

### Determination of Danshen–Rivaroxaban Interaction in RLM and HLM

The incubation system (200 μL) consisted of 0.05 mM Tris-HCl buffer (pH 7.4), RLM (1 mg/mL) or HLM (0.5 mg/mL), an NADPH-regenerating system including MgCl_2_ (5 mM), G6P (10 mM), G6PDH (0.4 U/mL), and NADP (1 mM), and rivaroxaban (20 μM). To determine the half-maximal inhibitory concentration (IC_50_), the concentration of Danshen tablet was set at 0.5, 1, 2, 5, and 10 mg/mL. To investigate whether the inhibition effects by Danshen tablet are time- and/or concentration-independent, the IC_50_ shift experiments containing RLM (1 mg/mL) or HLM (0.5 mg/mL), MgCl_2_ (5 mM), G6P (10 mM), G6PDH (0.4 U/mL), and 0.05 M Tris/HCl buffer (pH 7.4) with Danshen (0.5–10 mg/mL) were carried out under three different conditions: 0 min pre-incubation, 20 min pre-incubation plus NADPH, and 20 min pre-incubation minus NADPH.

To determine the mechanism underlying the inhibitory effect of Danshen tablet on rivaroxaban, the concentrations of Danshen tablet were set at 0.5, 1, 2, and 5 mg/mL and the concentration of rivaroxaban was set at 20, 30, 50, and 100 μM (according to the corresponding K_m_ value). Then, 1 mM of NADP was added to initiate the reaction. After incubation for 60 min, the reaction was immediately terminated by adding 200 μL ice-cold acetonitrile and 20 μL mebendazole (2 μg/mL), an internal standard, to the mixture. After vortexing for 3 min and centrifugation at 16,000 *g* for 15 min, the supernatant (80 μL) was transferred into the autosampler vial, and 2 μL was used for LC-MS/MS analysis.

### Inhibitory Effects of Danshen and Its Active Constituents on CYP1A2, CYP2B1, CYP2C11, CYP2D1, CYP2E1, CYP2J3, and CYP3A2 Activities in RLM

The incubation system (200 μL) consisted of 0.05 mM of Tris-HCl buffer (pH 7.4), pooled RLM (1 mg/mL), with Danshen tablet (2 mg/mL) or its main active ingredients (dihydrotanshinone I, tanshinone I, tanshinone IIA, cryptotanshinone, danshensu, salvianolic acid A, salvianolic acid B, and salvianolic acid C, 20 μM) and an NADPH-regenerating system. Substrates were added to the incubation mixture to obtain the final concentrations (10 μM phenacetin for CYP1A2, 20 μM bupropion for CYP2B1, 20 μM tolbutamide for CYP2C11, 5 μM dextromethorphan for CYP2D1, 20 μM chlorzoxazone for CYP2E1, 150 μM astemizole for CYP2J3, and 10 μM midazolam for CYP3A2). The proportion of the organic solvent was not higher than 1% (v/v) in the incubation mixture. Based on FDA guidelines, the CYP inhibition method was validated with positive inhibitors, and the results confirmed the effectiveness and reliability of the method. To determine the IC_50_ values, the concentration of Danshen tablet was set at 0.1–10 mg/mL, and the concentration of dihydrotanshinone I ranged from 0.5 to 50 μM. The mixture without NADP was pre-incubated at 37°C for 5 min. Then, 1 mM of NADP was added to initiate the reaction. After incubation for 20 min, the reaction was immediately terminated by adding 200 μL ice-cold acetonitrile and 20 μL mebendazole (2 μg/mL). The following processing steps are the same as the abovementioned experiments.

### Statistical Analysis

All data were presented as mean ± SD. The IC_50_ values were determined by a nonlinear regression analysis of plotting relative activities over the logarithm of inhibitor concentrations. Enzyme kinetics data were fitted with nonlinear regression analysis using GraphPad Prism 8.0 (GraphPad Software Inc., CA, United States). The data were fitted to the Michaelis–Menten model and further analyzed by using the Lineweaver–Burk plot (the reciprocal of reaction velocities versus the reciprocal of substrates concentrations). The Lineweaver–Burk plot was used to determine the quality of fit to a specific inhibition model. The inhibition constant (*K*_i_) was obtained by a secondary plot using the slopes of the primary Lineweaver–Burk plot (K_m_/V_max_ versus inhibitor concentration). The α*K*_i_ was obtained from a secondary plot using the y-intercepts of the Lineweaver–Burk plot (1/V_max_ versus inhibitor concentration). All results were analyzed in quadruplicate. One-way analysis of variance was used to estimate the significance of differences. Statistical significance was set at *p* < 0.05.

## Results

### Inhibitory Effect and Type of Danshen Tablet on Rivaroxaban Metabolism in RLM and HLM

**FIGURE 1:**
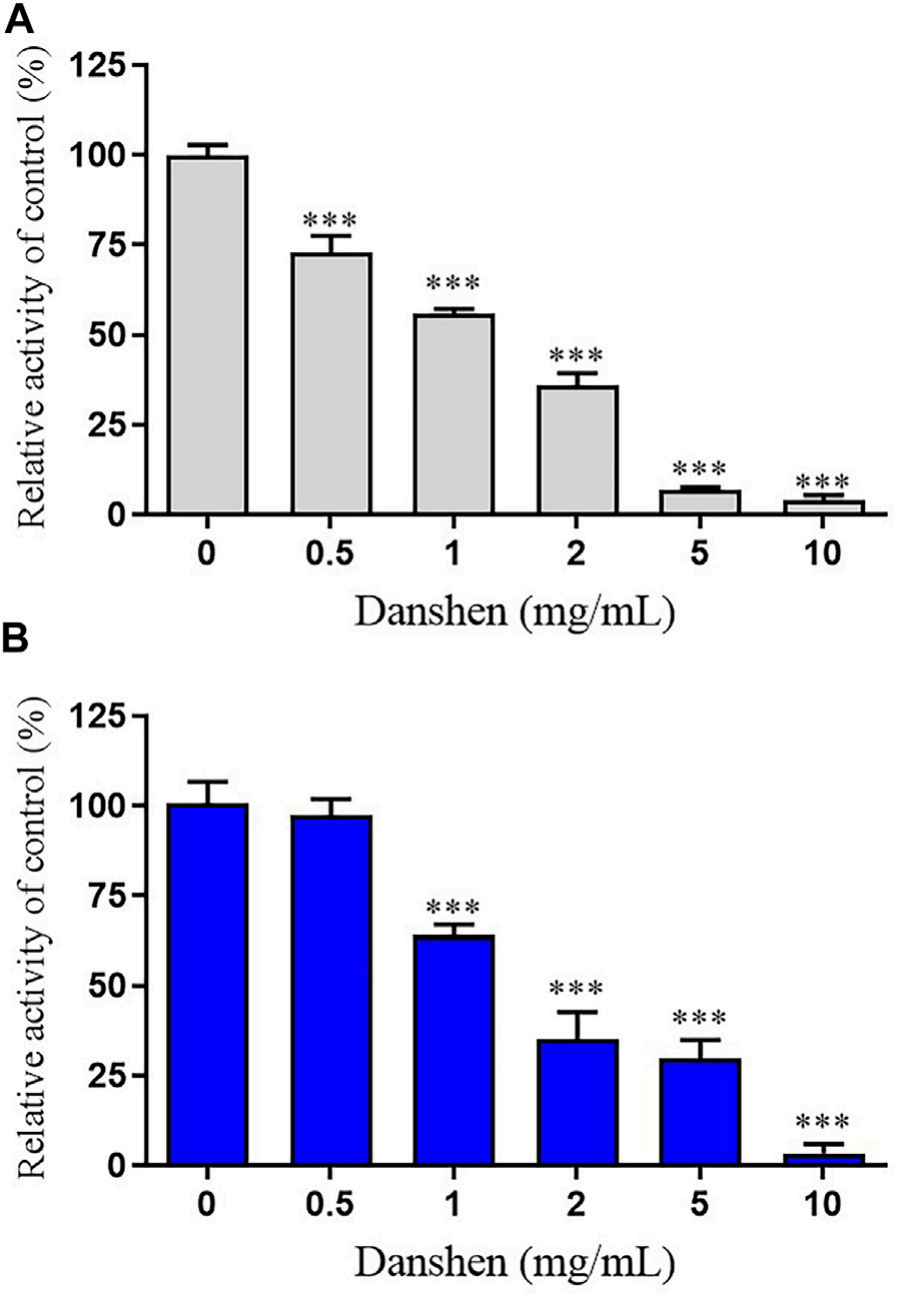
Inhibition of rivaroxaban metabolism by Danshen tablet in rat liver microsomes (RLM) **(A)** and human liver microsomes (HLM) **(B)**. Results were mean ± SD of quadruplicate determinations. ***p < 0.001 compared to control (no inhibitor).

Danshen tablet inhibited the rivaroxaban metabolism in a concentration-dependent manner (Figure 1). The IC_50_ value was further calculated, which showed that it was 1.16 and 1.85 mg/mL in RLM and HLM, respectively. To characterize the reversible or irreversible inhibition by Danshen tablet, the time- and concentration-independent experiments were used to determine whether it was both time-dependent and concentration-dependent. In the IC_50_ shift profiles, no significant changes in IC_50_ values were observed, thus suggesting Danshen tablet was a reversible inhibitor in RLM and HLM.

**FIGURE 2:**
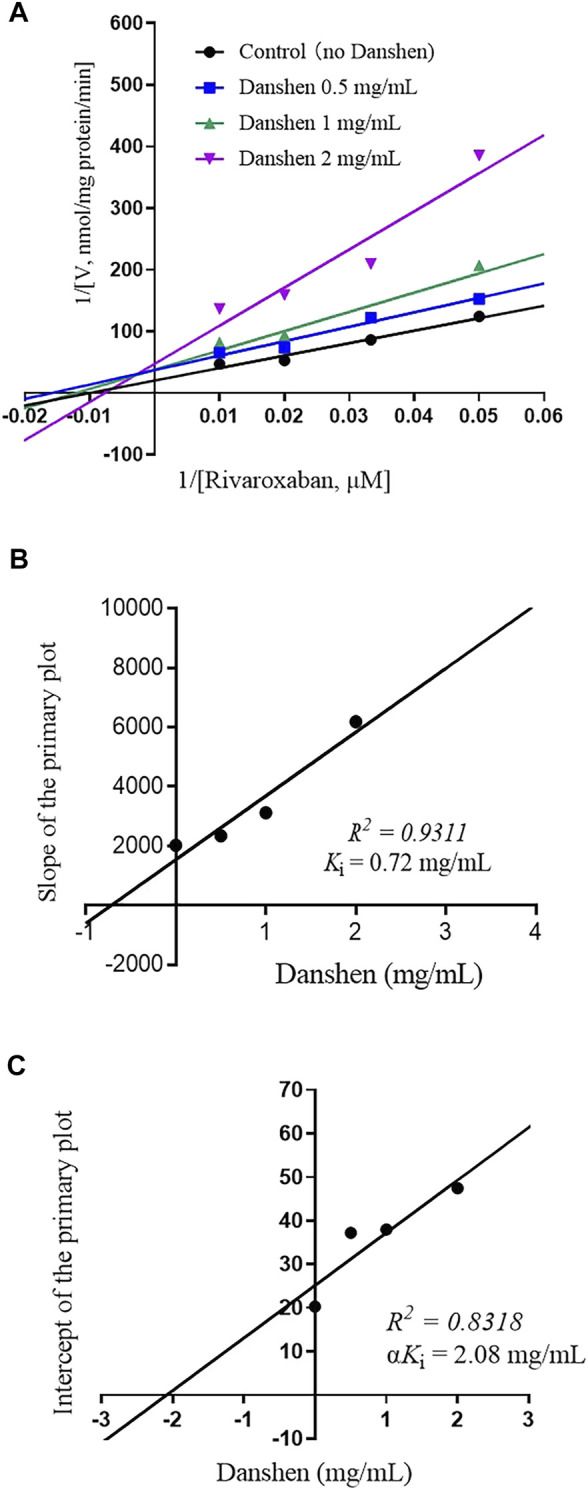
Primary Lineweaver–Burk plot **(A)**, the secondary plot for *K*_i,_ **(B)**, and the secondary plot for α*K*_i_ **(C)** for the inhibition of rivaroxaban metabolism by Danshen tablet with various concentrations (0.5, 1, and 2 mg/mL) in RLM. Rivaroxaban was used at 20, 30, 50, and 100 μM. Each data point represents the mean of quadruplicate determinations.

**FIGURE 3:**
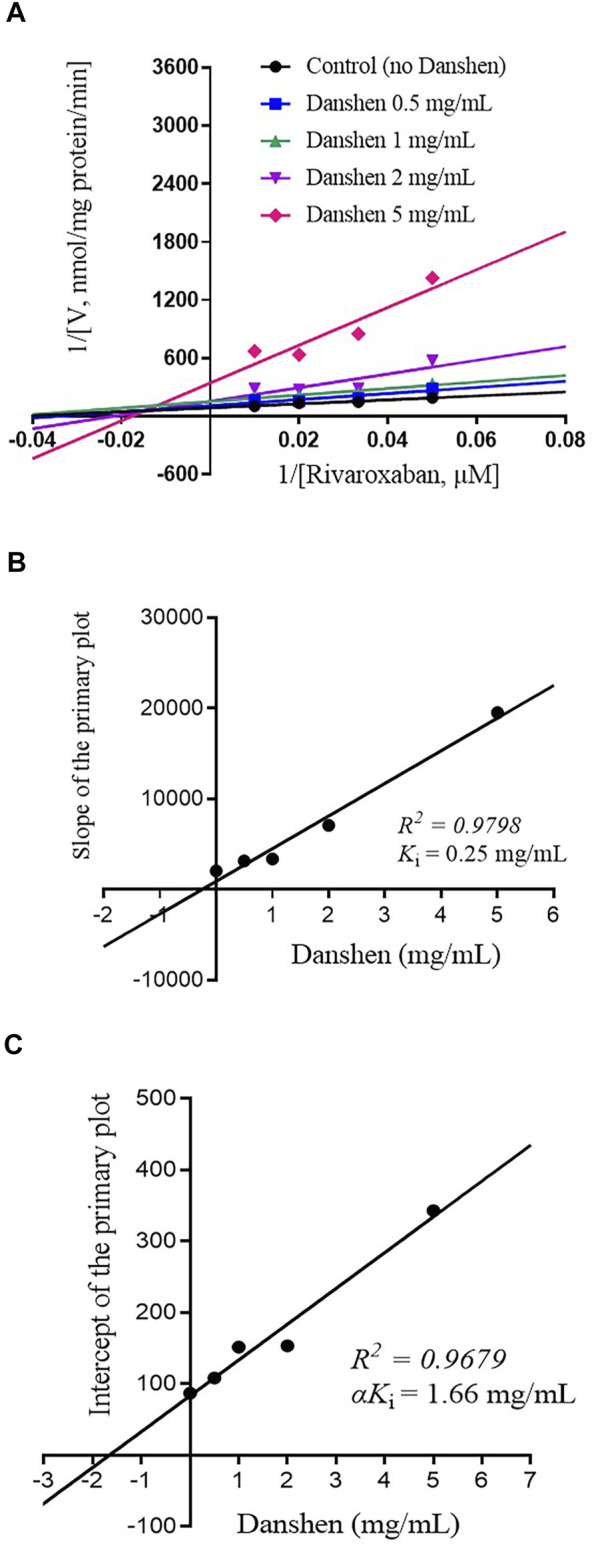
Primary Lineweaver–Burk plot **(A)**, the secondary plot for *K*_i_ **(B)**, and the secondary plot for α*K*_i_ **(C)** for the inhibition of rivaroxaban metabolism by Danshen tablet with various concentrations (0.5, 1, 2, and 5 mg/mL) in HLM. Rivaroxaban was used at 20, 30, 50, and 100 μM. Each data point represents the mean of quadruplicate determinations.

To further investigate the inhibitory mode of Danshen tablet, enzyme inhibition kinetic experiments were carried out with different concentrations of rivaroxaban (Figures 2A, 3A). The *K*_i_ values were determined by using the secondary Lineweaver–Burk plot and then calculated at 0.72 and 0.25 mg/mL in RLM (Figure 2B) and HLM (Figure 3B), respectively. From the secondary plot of Lineweaver–Burk plot for α*K*_i_, α*K*_i_ values were 2.08 and 1.66 mg/mL in RLM (Figure 2C) and HLM (Figure 3C), respectively. Since α values (2.89 in RLM and 6.64 in HLM) were not equal to 1, the type of inhibition for Danshen tablet was mixed inhibition.

### Inhibitory Effects of Danshen Tablet and Its Active Components on CYP Activities in RLM

**FIGURE 4:**
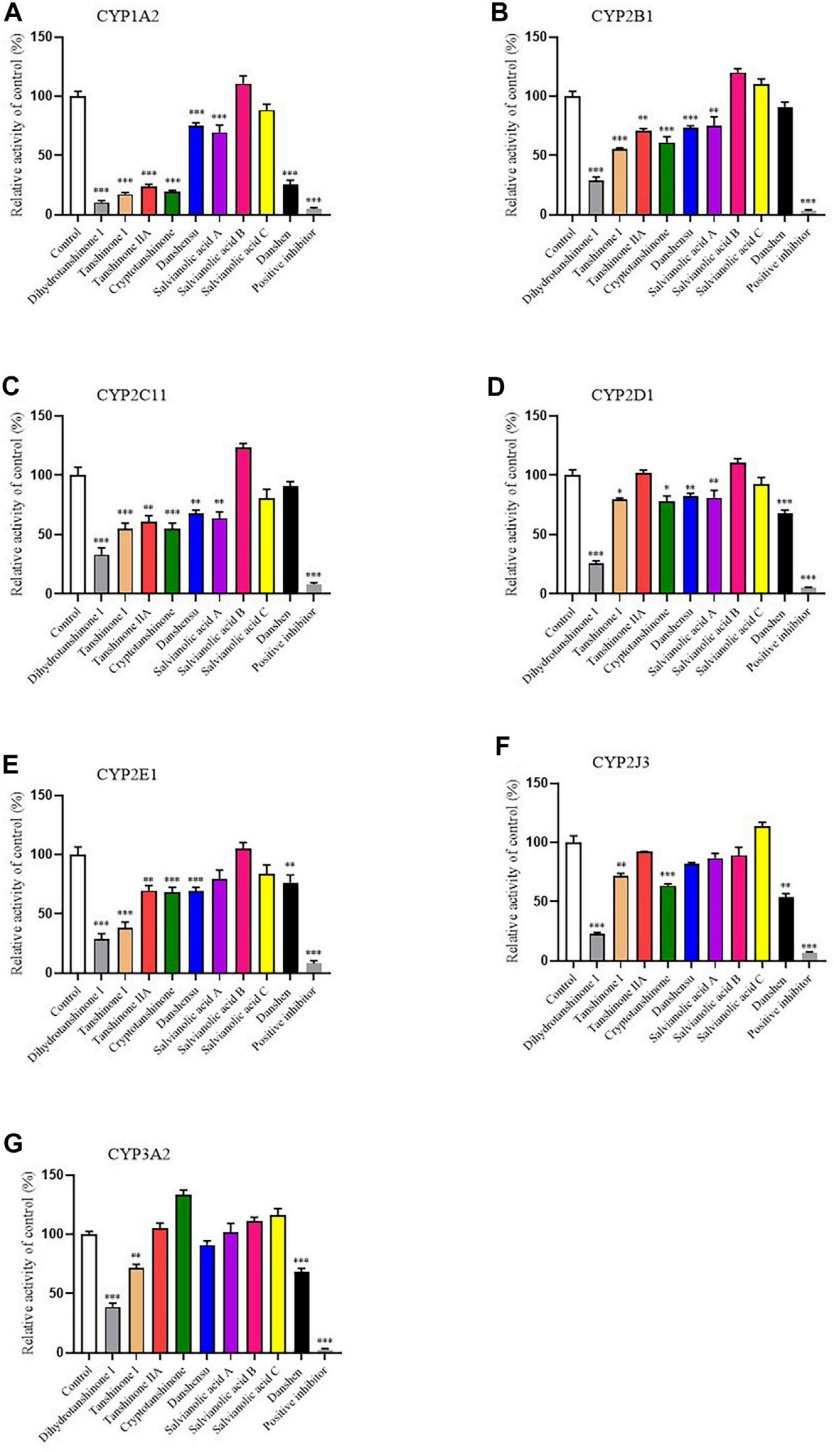
Inhibitory effects of eight active components of Danshen (dihydrotanshinone I, tanshinone I, tanshinone IIA, cryptotanshinone, danshensu, salvianolic acid A, salvianolic acid B, and salvianolic acid C, 20 μM) and Danshen tablet (2 mg/ml) on activities of CYP1A2 **(A)**, CYP2B1 **(B)**, CYP2C11 **(C)**, CYP2D1 **(D)**, CYP2E1 **(E)**, CYP2J3 **(F)**, and CYP3A2 **(G)** in RLM. Positive inhibitors included furafylline (CYP1A2), thiotepa (CYP2B1), sulfaphenazole (CYP2C11), quinidine (CYP2D1), disulfiram (CYP2E1), flunarizine (CYP2J3), and ketoconazole (CYP3A2). Values are presented as mean ± SD (*n* = 4). *p < 0.05, **p < 0.01, and ***p < 0.001 compared to control (no inhibitor).

In order to study the effects of Danshen tablet and its main components on the activities of important CYP subtypes, we chose medium-concentration of Danshen tablets (2 mg/mL) and its main active ingredients (20 μM) to carry out the screening assay. The data showed that Danshen tablet had inhibitory effects on CYP1A2, CYP2D1, CYP2E1, CYP2J3, and CYP3A2 (Figure 4). Lipid-soluble components, such as tanshinone I, tanshinone IIA, dihydrotanshinone I, and cryptotanshinone, generally have stronger inhibitory effects on CYP enzymes than aqueous soluble components, such as danshensu and salvianolic acid A, B, and C. Among all components, dihydrotanshinone I showed the strongest inhibition on all CYP subtypes (Figure 4), indicating that it is the main component inhibiting CYP enzyme activity in Danshen tablet.

**FIGURE 5:**
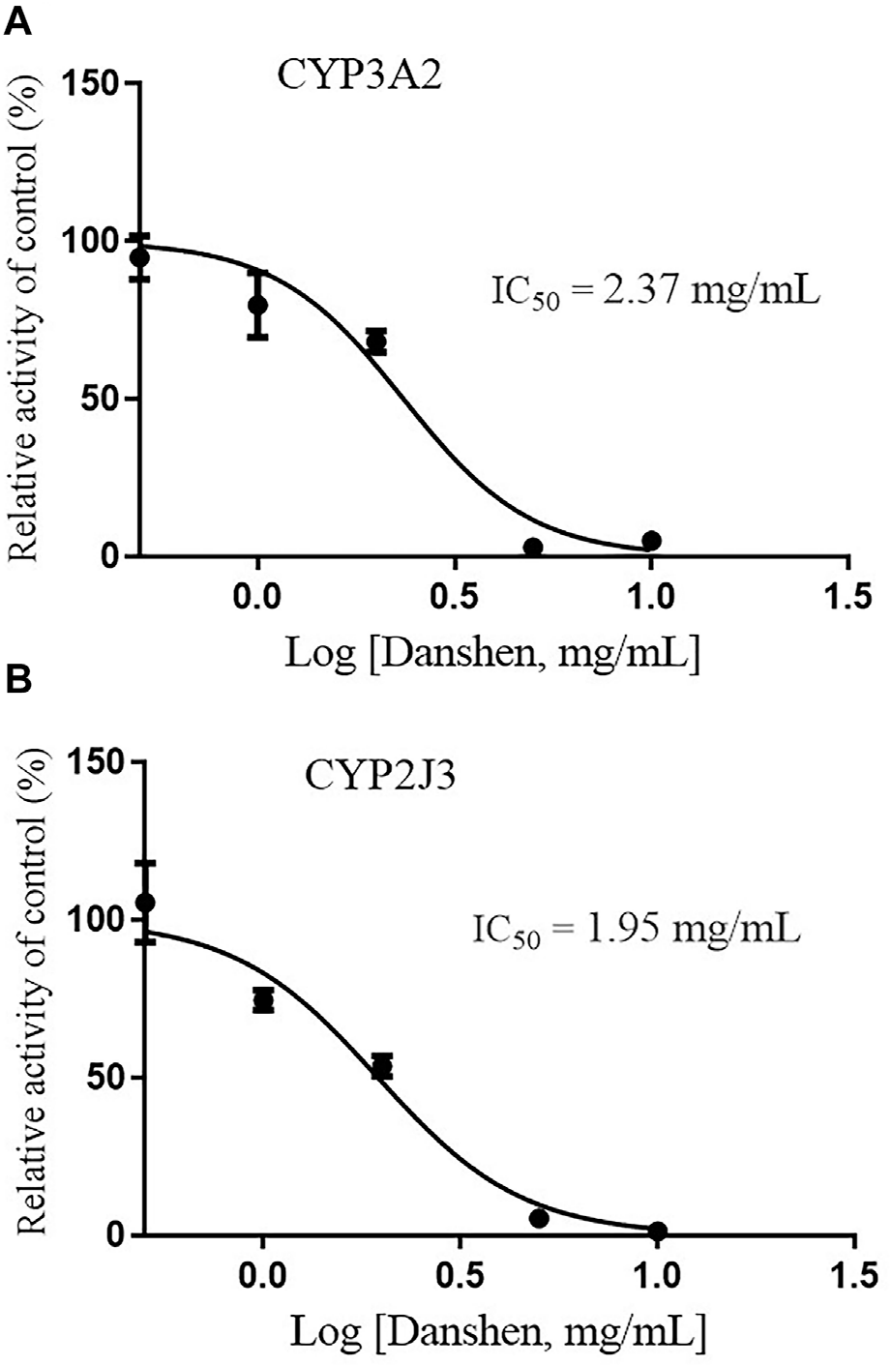
Inhibition of CYP3A2 **(A)** and CYP2J3 **(B)** activities by Danshen tablet (0.5–10 mg/mL) in RLM. Values are expressed as mean ± SD of quadruplicate assays. The data were fit to log (Danshen concentration) and normalized response equations using GraphPad Prism 8.0.

**FIGURE 6:**
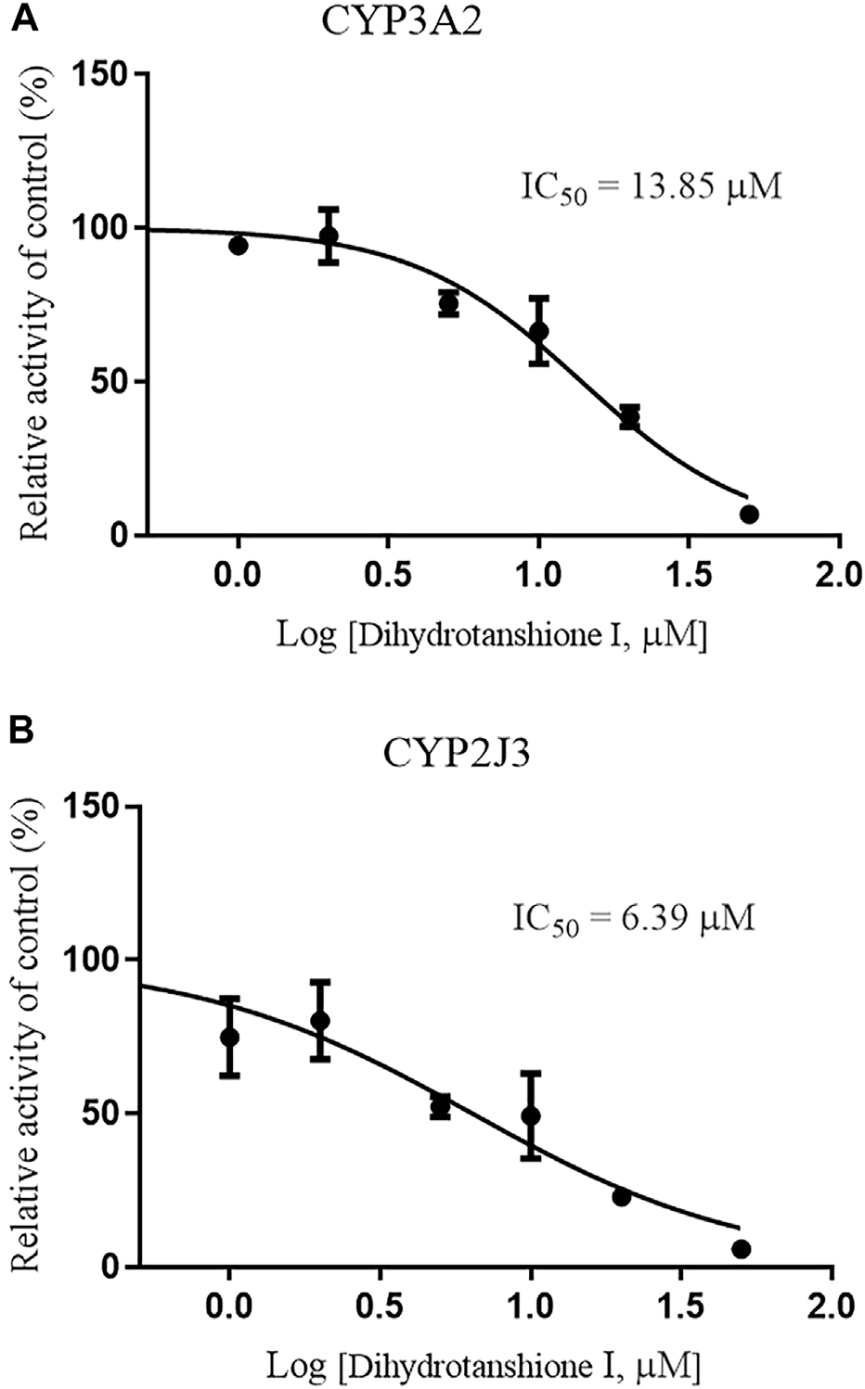
Inhibition of CYP3A2 **(A)** and CYP2J3 **(B)** activities by dihydrotanshione I (1–50 μM) in RLM. Values are expressed as mean ± SD of quadruplicate assays. The data were fit to log (dihydrotanshione I concentration) and normalized response equations using GraphPad Prism 8.0.

Since CYP3A4 and CYP2J2 are the main CYP enzymes mediating rivaroxaban metabolism in humans (Cheong et al., 2017; Wang et al., 2021), we further detected the value of IC_50_ values of Danshen tablet and dihydrotanshinone I on CYP3A2 and CYP2J3 in RLM. The results indicated that Danshen tablet inhibited CYP3A2 and CYP2J3 activities in a concentration-dependent manner, with IC_50_ values of 2.37 and 1.95 mg/mL, respectively (Figure 5). In addition, dihydrotanshinone I had a strong inhibitory effect on CYP3A2 and CYP2J3 activities, with IC_50_ values of 13.85 and 6.39 μM, respectively (Figure 6).

## Discussion

Cardiovascular disease (CVD) is the leading cause of morbidity and mortality around the world (Zeng et al., 2022). Cardiac arrhythmias are the abnormalities of heartbeat. Atrial fibrillation (AF) is the most common cardiac rhythm disorder in hospitals, accounting for one-third of arrhythmia-related hospitalizations (Chen et al., 2019). Non-valvular AF (NVAF) is the most common form of AF with severe complications (Chen et al., 2019). Oral anticoagulants (OAC) reduce the risk of stroke and death caused by NVAF (Chen et al., 2019). OAC includes vitamin K antagonists (VKA) and non-VKA oral anticoagulants (NOAC), which inhibit activating factor Xa or thrombin (Chen et al., 2019; Purrucker et al., 2020). Factor Xa plays an important role in both intrinsic and extrinsic coagulation pathways as it leads to thrombin activation (Chen et al., 2019). The current guidelines of the European Heart Rhythm Association (EHRA) suggest that NOAC is not recommended in combination with drugs that are strong inhibitors of CYP3A4. Inhibition of CYP3A4 may affect the excretion and metabolism of NOAC, resulting in an increased risk of massive bleeding admission (Steffel et al., 2018).

Rivaroxaban, as a small-molecule inhibitor of factor Xa, can reduce the risk of recurrence of cardiovascular events. It contributes to the treatment of cardiovascular diseases, especially ACS and AF (Chen et al., 2019). Rivaroxaban combined with verapamil (inhibitor of CYP3A4 and P-gp) may be unsafe and lead to CRNM bleeding (Sychev et al., 2020). Traditional Chinese medicine (TCM) is used to prevent and treat CVD, and Danshen is a golden herbal medicine in the treatment of CVD (Wang L. et al., 2017; Li et al., 2018). Clinical studies have found that more than one-third of patients with cardiovascular disease have taken both drugs and herbs in the past 12 months (Yeh et al., 2006). Due to the diversity and complexity of herbal components and the lack of understanding of the pharmacokinetics of pathogenic components, the mechanism of interaction between herbal medicine and drugs is still unknown to a large extent. Therefore, it is necessary to further evaluate the HDI between herbs and anticoagulants in NVAF patients, especially drug-metabolizing enzymes involved. Based on the interaction mediated by CYP enzymes, this study investigated the potential interaction between Danshen tablet and rivaroxaban, which may be related to the side effects of rivaroxaban. Our results showed that Danshen tablet had a strong inhibitory effect on rivaroxaban metabolism in human and rat liver microsomes. Further inhibition kinetic study showed that the Danshen tablet was a mixed inhibitor with *K*_i_ values at 0.72 and 0.25 mg/mL in rat and human liver microsome, respectively. These data directly show that there is an interaction between Danshen tablet and rivaroxaban, and Danshen tablet inhibits rivaroxaban metabolism.

As the most important phase I drug metabolism enzyme, CYP is involved in catalyzing the metabolism of most clinical drugs (Li et al., 2019). In humans, the main CYP subtypes include CYP1A2, CYP2B6, CYP2C9, CYP2D6, CYP2E1, CYP2J2, and CYP3A4 (Tang et al., 2017). These subtypes participate in the metabolism of more than 90% of prescription drugs and play an important role in clinical practice (Ouyang et al., 2019). Inhibition or induction of CYP enzyme activity is the main cause of drug–drug or herb–drug interactions (Chen et al., 2016). Therefore, we carried out the CYP activity screening experiments on Danshen tablet and its eight main pharmacological active components. The results showed that Danshen tablet and its lipid-soluble components, such as tanshinone I, tanshinone IIA, and dihydrotanshinone I had potently inhibitory effects on CYP1A2, CYP2B1, CYP2C11, CYP2E1, CYP2J3, and CYP3A2. Our results are consistent with the data reported in previous studies. For example, tanshinone I, and tanshinone IIA are competitive inhibitors of CYP1A2, CYP2C9, and CYP2E1, but the inhibition of CYP3A4 is weak (Wang et al., 2010). Dihydrotanshinone potently inhibits CYP1A2, CYP2C9, and CYP3A4 in human liver (Wang et al., 2010).

CYP3A4 is the most important human drug-metabolizing enzyme involved in drug metabolism (Sun et al., 2015; Li et al., 2019). At the same time, CYP3A4 is also an enzyme that maintains the homeostasis of endogenous substances (Qin and Wang, 2019). For example, CYP3A regulates bile acid (BA) homeostasis in rats and prevents hepatotoxicity caused by BA overload (Qin et al., 2021). CYP2J2 is a major cardiac CYP enzyme, which mainly metabolizes polyunsaturated fatty acids (Das et al., 2020). CYP2J2 plays an important role in CVD (Azevedo et al., 2016). For example, CYP2J2 transgenic mice have a reduced rate of arrhythmia induction (Westphal et al., 2013). Our previous study also found that knockout of CYP2J3/10 in rats led to heart disease (Lu et al., 2020). In addition, CYP2J2*7 allele has been reported to be associated with stroke and CVD (Wang SY. et al., 2017). It is worth noting that human CYP3A4 and CYP2J2 are the main enzymes involved in rivaroxaban metabolism (Cheong et al., 2017; Wang et al., 2021). Our results show that Danshen tablet and tanshinones, especially dihydrotanshinone I, can effectively inhibit the activities of CYP3A and CYP2J enzymes. Therefore, the interaction between Danshen and rivaroxaban probably occurs because Danshen and its tanshinones inhibit CYP3A and CYP2J activities and then inhibit the metabolism of rivaroxaban. Further *in vivo* experiments such as pharmacodynamics and pharmacokinetics (PD-PK) of Danshen and rivaroxaban are needed to study the interactions between Danshen and rivaroxaban and substantiate their internal mechanism.

In conclusion, the combination of Danshen tablet and rivaroxaban may have potential risks. Danshen and tanshinones may inhibit the metabolism of rivaroxaban by inhibiting CYP3A and CYP2J activities. In the follow-up study, the pharmacokinetics and pharmacodynamics of Danshen and rivaroxaban need to be tested *in vivo*, and more rigorous clinical trials in humans should be considered to verify this finding.

## Data Availability

The original contributions presented in the study are included in the article/Supplementary Material; further inquiries can be directed to the corresponding authors.

## Abbreviations

HDI, herb–drug interaction; CYP, cytochrome P450; DOAC, direct oral anticoagulant; ACS, acute coronary syndrome; NVAF, non-valvular atrial fibrillation; HLM, human liver microsomes; RLM, rat liver microsomes; IC_50_, half-maximal inhibitory concentration; Ki, inhibition constant.
