# Corrigendum: Evaluation of herb–drug interaction between Danshen and rivaroxaban in rat and human liver microsomes

**DOI:** 10.3389/fphar.2022.1039267

**Published:** 2022-10-05

**Authors:** Xu Wang, Jingjing Fa, Yuanjin Zhang, Shengbo Huang, Jie Liu, Junqing Gao, Lina Xing, Zongjun Liu, Xin Wang

**Affiliations:** ^1^ Department of Cardiology, Putuo Hospital, Shanghai University of Traditional Chinese Medicine, Shanghai, China; ^2^ Shanghai Key Laboratory of Regulatory Biology, Institute of Biomedical Sciences, School of Life Sciences, East China Normal University, Shanghai, China; ^3^ Putuo Clinical Medical School, Anhui Medical University, Shanghai, China

**Keywords:** Danshen, rivaroxaban, herb-drug interaction, metabolism, cytochrome P 450 (CYP)

In the published article, there was an error in affiliation 1. Instead of “Department of Cardiology, Central Hospital of Shanghai Putuo District, Shanghai University of Traditional Chinese Medicine, Shanghai, China”, it should be “Department of Cardiology, Putuo Hospital, Shanghai University of Traditional Chinese Medicine, Shanghai, China”.

The authors apologize for this error and state that this does not change the scientific conclusions of the article in any way. The original article has been updated.

